# High-throughput method for ear phenotyping and kernel weight estimation in maize using ear digital imaging

**DOI:** 10.1186/s13007-018-0317-4

**Published:** 2018-06-15

**Authors:** R. Makanza, M. Zaman-Allah, J. E. Cairns, J. Eyre, J. Burgueño, Ángela Pacheco, C. Diepenbrock, C. Magorokosho, A. Tarekegne, M. Olsen, B. M. Prasanna

**Affiliations:** 1 International Maize and Wheat Improvement Center (CIMMYT), PO Box MP163, Harare, Zimbabwe; 2 University of Queensland, Brisbane, Australia; 3 International Maize and Wheat Improvement Center (CIMMYT), PO Box 1041, Nairobi, Kenya; 4 International Maize and Wheat Improvement Center (CIMMYT), El Batan, Mexico; 5 Cornell University, Ithaca, NY 14853 USA

**Keywords:** Maize, Ear, Kernel, Phenotyping, Image analysis

## Abstract

**Background:**

Grain yield, ear and kernel attributes can assist to understand the performance of maize plant under different environmental conditions and can be used in the variety development process to address farmer’s preferences. These parameters are however still laborious and expensive to measure.

**Results:**

A low-cost ear digital imaging method was developed that provides estimates of ear and kernel attributes i.e., ear number and size, kernel number and size as well as kernel weight from photos of ears harvested from field trial plots. The image processing method uses a script that runs in a batch mode on ImageJ; an open source software. Kernel weight was estimated using the total kernel number derived from the number of kernels visible on the image and the average kernel size. Data showed a good agreement in terms of accuracy and precision between ground truth measurements and data generated through image processing. Broad-sense heritability of the estimated parameters was in the range or higher than that for measured grain weight. Limitation of the method for kernel weight estimation is discussed.

**Conclusion:**

The method developed in this work provides an opportunity to significantly reduce the cost of selection in the breeding process, especially for resource constrained crop improvement programs and can be used to learn more about the genetic bases of grain yield determinants.

## Background

In maize, yield is a function of interdependent characteristics of ears and kernels [1]. A well-developed maize ear may have close to a thousand kernels [2]. The number of kernels per ear is a function of ear width (kernels per row) and kernel rows per ear. Many stresses can affect row number and kernels per row, as well as kernel size/weight. Cairns et al. [3] reported that under drought conditions, yield loss in both hybrids and inbreds was largely associated with a highly significant decrease in the number of kernels per unit of ear area. Plant water deficit at flowering has been shown to negatively affect kernel number [4] and deficiencies in N supply usually decrease grain yield by lowering kernel number per plant [5, 6] as a result of less synchronous pollination [7], and/or greater kernel abortion [8]. This indicates that these ear and kernel features can be used to assess the tolerance of a variety to a stressful condition. From a breeding perspective, studies have found that yield components tend to display greater heritability than overall yield [9, 10]; making it possible to select for these traits separately and then combine the responsible genetic loci to develop a genotype with superior performance or develop a selection index through traits combinations [11]. According to Miller et al. [1], if maize ears, and kernels attributes could be automatically measured with greater objectivity and precision, more could be learned about the genetic bases of yield components and how to improve them using current and future maize genetic resources.

There are few methods that allow the extraction of ear and kernel features through image processing. A method of evaluating one or more kernels of an ear of maize using digital imagery was patented by Pioneer (Hi-Bred International, inc., Iowa) in 2009 [12]. The method enables to extract kernel count, kernel size distribution, proportion of kernels aborted and other information using image processing algorithms that include, without limitation, filtering, watershedding, thresholding, edge finding, edge enhancement, color selection and spectral filtering. Zhao et al. [13] have proposed a method that provides kernel counts from ear photos, with the assumption that a maize ear has double the number of rows and kernels than can be visible on a photo. More recently, Liang et al. [14], have developed a method that scores maize kernel traits based on line-scan imaging. The method provides 12 maize kernel traits through image processing under controlled lighting conditions. In addition, Miller et al. [1] have proposed three custom algorithms designed to compute kernel features automatically from digital images acquired by a low cost platform. One algorithm determines the average space each kernel occupies along the cob axis using a sliding-window Fourier transform analysis of image intensity features. The second one counts individual kernels removed from ears, including those in clusters. The third one measures each kernel’s major and minor axis. The main limitation of these methods is that they often rely on systems like a scanner that have controlled lighting conditions and fixed image background. In addition, they do not provide a comprehensive data set from a single image of unthreshed ears i.e. ear count, ear and kernel features simultaneously in an automated manner.

Although there are harvesting equipments that automatically measure grain yield on a plot level, yield component traits such as ear and kernel dimensions are usually measured by hand [15–17]. In addition, this kind of equipment is quite expensive to buy and maintain, therefore not affordable for most breeding programs, especially in sub-Saharan Africa. Digital imaging provides a rapid and low-cost option to collect a large number of ear related traits and has the potential to improve our ability to evaluate yield potential in a breeding program and ultimately help characterize maize lines and advance our understanding of the genetic mechanisms controlling the fundamental yield components [1].

This work reports a simple, high-throughput and robust method for extracting yield components (ear and kernel attributes) from harvested maize ears using ear digital imaging (EDI).

## Materials and methods

### Germplasm and experiments

The study was conducted at CIMMYT research station (17°43′37.21′′ S, 31°01′00.60′′ E, and altitude 1489 m above sea level) in Harare, Zimbabwe.

**Fig. 1:**
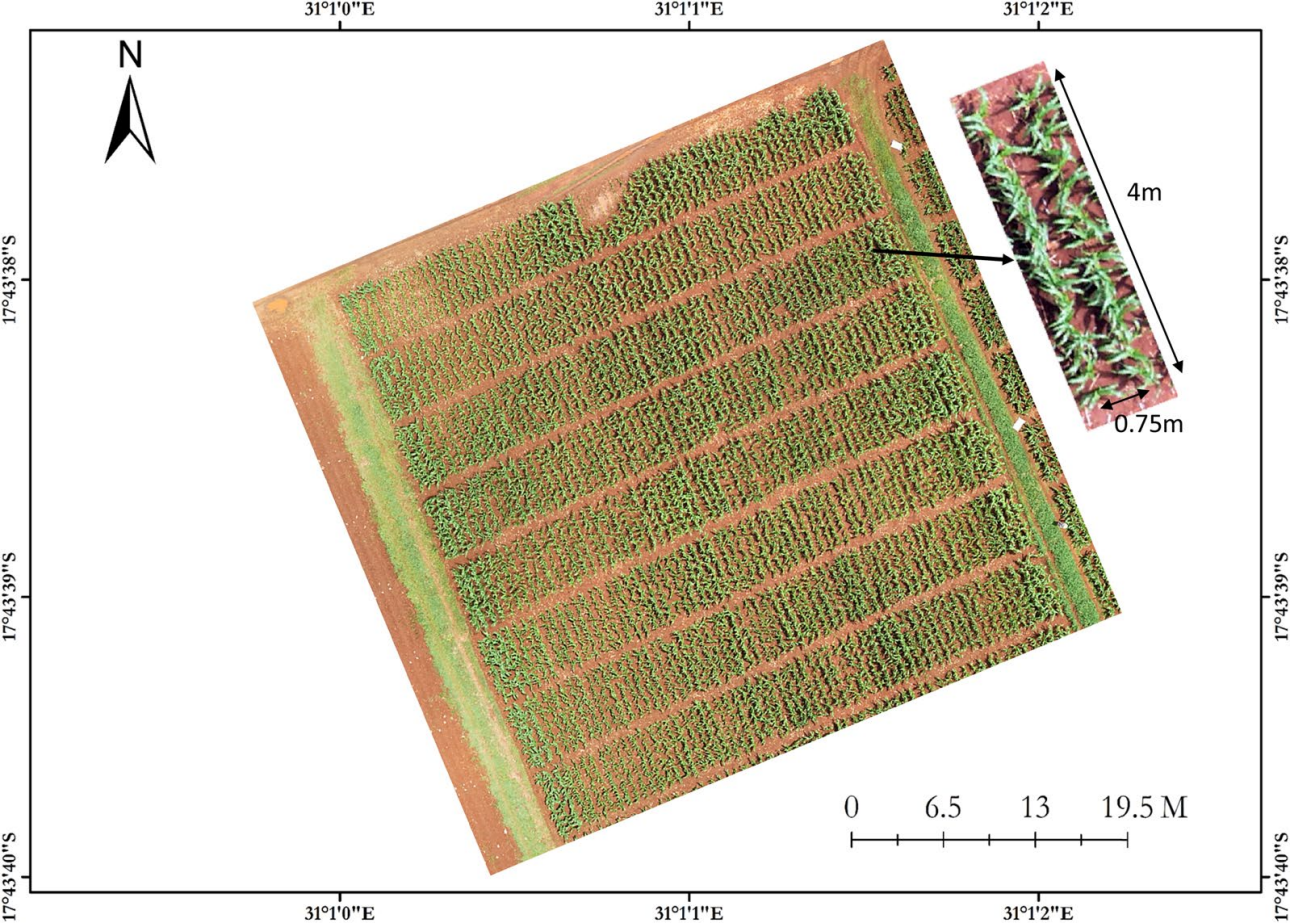
Overall view of the experimental setup and single plot details

*Development of kernel count and weight models* To develop these models, one trial composed of 10 hybrids was planted in two replicates on 3 December 2015 using an alpha lattice design. Each hybrid was represented by 2-row plots that were 4 m long with inter-row spacing of 0.75 m and in-row spacing of 0.25 m (Fig. 1). Each plot had approximately 34 plants. After physiological maturity, the ears were collected from each plot separately and dried to approximately 10–12% kernel moisture content.

*Validation of the EDI method for ear and kernel count and size* The validation was performed using one trial composed of 50 hybrids that were planted in three replicates on 15 December 2016 using an alpha lattice design with a total of 150 plots. The plots specifications were the same as described above. At harvest, the ears were selected from different ear sizes so as to cover as much as possible a large range of sizes.

*Validation of kernel weight model and heritability of traits* To validate the kernel weight model and assess the broad-sense heritability of ear traits generated through EDI, a total of six breeding trials were planted on 15 December 2016 using an alpha lattice design. They were composed of advanced elite and pre-commercial sub-tropical maize hybrids which were separated into three maturity groups; early, intermediate and late based on the number of days to flowering. Four of the trials had 50 hybrids each and the remaining two were composed of 55 hybrids each. Trials were all under low nitrogen stress. The plot specifications were the same as described above. Therefore, each trial with 50 hybrids had a total of 150 plots while those with 55 hybrids had 165 plots. For each plot, the ears were collected from all plants after physiological maturity.

### Photo acquisition

**Fig. 2:**
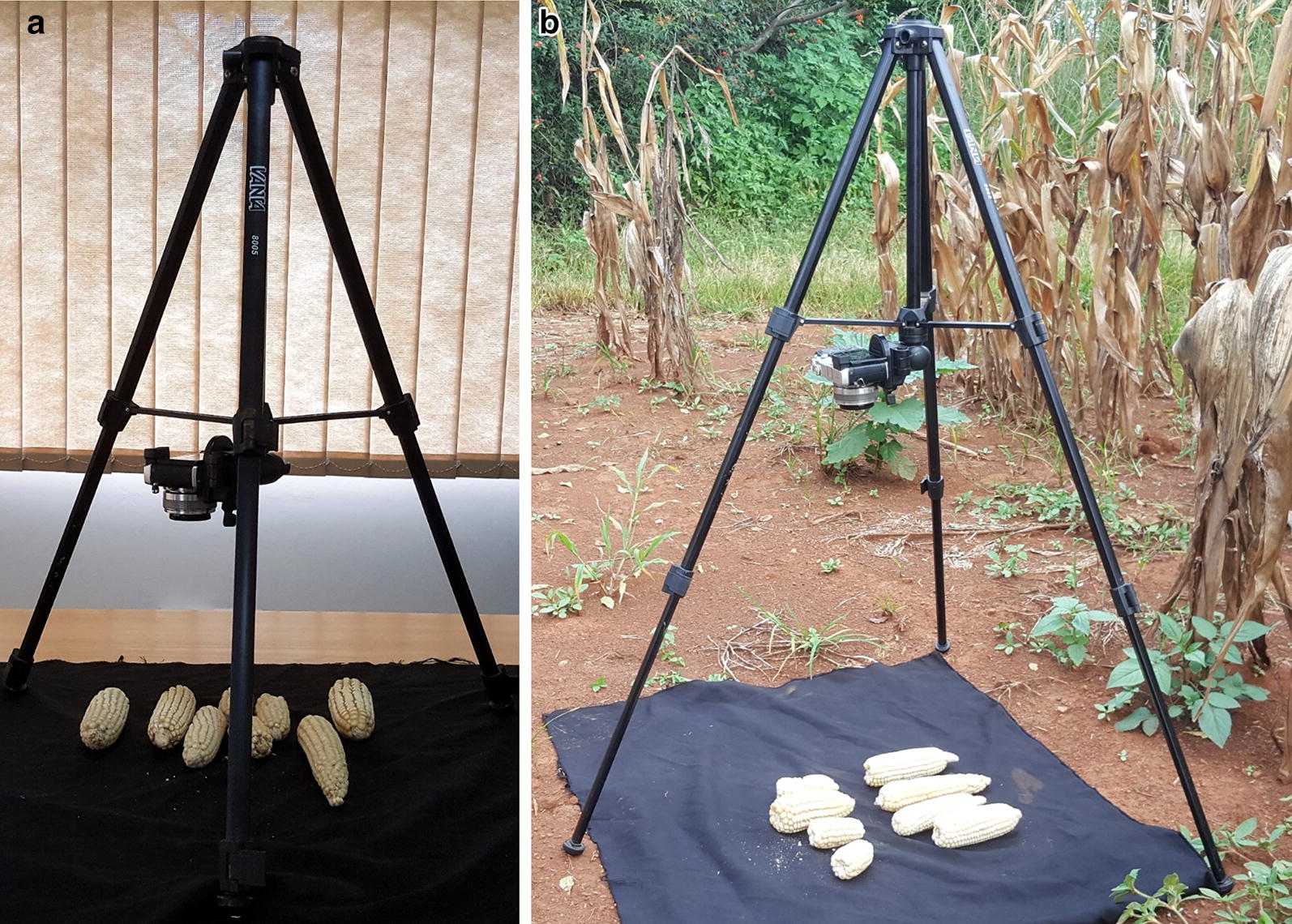
Photo acquisition set up under **a** diffuse lighting conditions and **b** field conditions

Ears were collected from field trials, de-husked and kept per plot. They were arranged on a black piece of cloth side by side in a way that they are not in much contact with each other. Digital photographs of all ears belonging to a plot were taken using a Sony camera (Cyber-shot DSC-WX80, 16.2 megapixels) set in automatic mode. The camera was mounted on a tripod stand at a height of 80 cm from the camera lens to the ground surface and positioned at nadir. For photo acquisition under controlled conditions, the set up was done in a room with diffuse lighting conditions (Fig. 2a). At the same height, an image with a ruler was also taken to convert the pixel scale measurements to centimentres. Similary, images for validation were also taken per plot under field conditions using a similar set up (Fig. 2b).

### Image processing

**Fig. 3:**
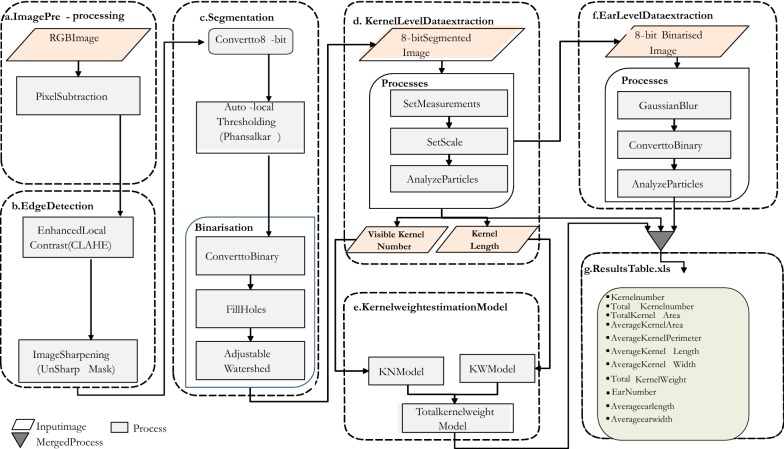
Workflow diagram of the image processing procedure

**Fig. 4:**
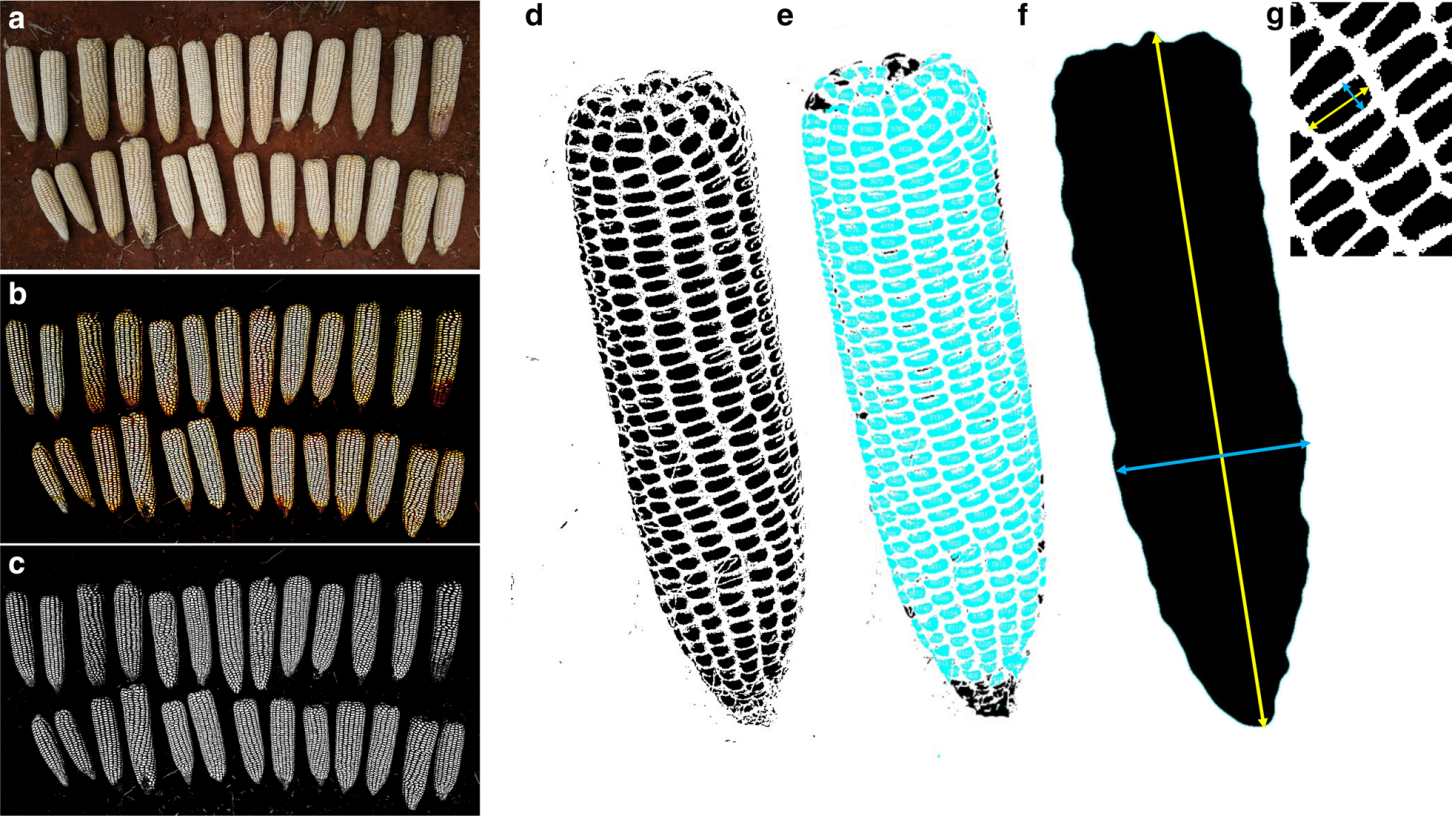
Example of images unfolding the image processing and data extraction key steps. **a** original image, **b** pre-processing step, **c** transformation into 8-bit, **d** binarization, **e** particles analysis, **f** ear attributes extraction, **g** kernel attributes extraction. Image **a** represents 1 plot under field conditions

Image analysis was conducted in imageJ [18], an open source software. Figure 3 shows a series of steps that were performed to segment and extract yield components parameters (i.e. ear and kernel attributes). These steps were performed using ImageJ plugins. An image pre-processing step was firstly carried out to distinctively separate the foreground (ears) from the background objects. Although there were many different ways to achieve this, a single image pixel subtraction method was used, which deducts a constant pixel value from an image. The pixel subtraction threshold was set to 100 based on tests carried out with 20 selected images contrasting for illumination gradient of the background to prevent foreground information loss. As a result, an image with an uniformly darker background intensity was produced (Fig. 4b). In this way, background pixels with same color intensities as kernels were suppressed minimizing possibility of significant noise during segmentation. Kernels are separated from one another by lines in between them over narrow colour gradients with fuzzy boundaries. The extent of boundary fuzziness and other surface artefacts of different nature could result in distortion of kernel edges owing to segmentation problems. Segmentation of kernels is primarily based on the clear definition of these edges whilst minimizing the effects of artefacts on their surfaces. Consequently, contrast limited adaptive histogram equalization (CLAHE) method was implemented to enhance the kernel edges whilst suppressing surface noise [19]. Unlike ordinary adaptive histogram equalization (AHE), which maps a narrow range of input intensity values on a wider range of output intensities values leading to over-enhancement of noise, with CLAHE, a maximum count of intensities can be enforced to limit the enhancement thereby reducing noise [19]. Whilst there is no enhancement at intensity value of 1, an increase in intensity levels subsequently increases enhancement. CLAHE is a well-known block-based processing, and it can overcome the over amplification of noise problem in the homogeneous region of image with standard histogram equalization.

The CLAHE plugin has three parameters. (i) Block size, which defines the size of the local region around a pixel for which the histogram is equalized, was set to 29, (ii) the number of histogram bins used for histogram equalization set to 256. The implementation internally works with byte resolution, so values larger than 256 are not meaningful. Then the maximum slope, which limits the contrast stretch in the intensity transfer function, was set to 5 (the value 1 will not result in any change in the original image). Enhanced edges were then sharpened to increase their intensity levels using the unsharp mask method with a radius of 5 and a mask set to 0.70. The image was then converted to 8-bit format.

Suppression of artefacts of low contrast was not completely dealt with at edge detection. A local threshold method by Phansalskar [20], a modification of Sauvola’s [21] method was used, which proved to be more effective in cytological images of low contrast. The threshold T(x, y) is calculated according to Eq. (1), where m(x, y) is the mean and s(x, y) standard deviation of pixel intensities, R is the dynamic range of standard deviation which is equal to 0.5 for normalized images, k constant in the range (0.2–0.5), q and p are Phansalkar’s exponential constants.

1 $$T\left( {x,y} \right) = m\left( {x,y} \right)\left[ {1 + pe^{{ - q.m\left( {x,y} \right)}} + k\left( {\frac{{s\left( {x,y} \right)}}{R}} \right) - 1} \right]$$

In the Phansalkar’s plugin, k and r are referred as parameters 1 and 2 respectively. They were kept to default values k = 0.25 and r = 0.5 which worked very well across ear types.

The radius of the local domain over which the threshold will be computed was set to 15. The white object on black background option was selected to set to white the pixels with values above the threshold value (otherwise, it sets to white the values less or equal to the threshold).

The images were then binarized with filling of holes to achieve solid kernel shapes which prevents splitting during the watershed step (Fig. 4c, d). An adjustable watershed plugin which provides flexibility through a wide range of tolerance levels to suit different kernel edge smoothness and shapes was applied with a tolerance of 3. The tolerance value determines the difference of radius between the smaller of the largest inscribed circles and a circle inscribed at the neck between the particles. The higher this value, the fewer segmentation lines and low values tend to produce false segmentations, caused by the pixel quantization. In this way kernel segmentation was successfully performed with minimum errors. The computational workflow is able to estimate yield components parameters (number of ears, size, kernel number and size) from approximately six images or plots per minute.

#### Kernel counts and attributes

The segmented images were then used for particles analysis after setting the minimum and maximum pixel area size (0.03–1.0 pixels^2^) to exclude anything that is not an object of interest in the image. In addition, circularity values were set within the interval 0.15–1.00 to help excluding unwanted objects with a value of 1.0 indicating a perfect circle. Circularity is a shape descriptor (https://imagej.nih.gov/ij/docs/guide/146-30.html). As the value approaches 0.0, it indicates an increasingly elongated shape. Kernel length and width are referred here as the longest distance between two points along the major and minor axis on a single kernel on the ear, respectively (Fig. 4g). In addition, the total kernel area, as the sum of all individual kernel areas on the image, and the average kernel area were generated using particle analysis. The average perimeter represents the average length of the outside boundary of all kernels that are on the analyzed image. Qualitative attribute like kernel color and ear texture were not included as they can be identified easily from visual observation.

#### Ear count and attributes

 For the ear count, kernels were filtered out via a Gaussian blur method. This filter uses convolution with a Gaussian function for smoothing (https://imagej.nih.gov/ij/docs/guide/146-29.html#sub:Gaussian-Blur). The parameter sigma was set to 10. Sigma is the radius of decay to exp(−0.5), (≈ 61%), i.e., the standard deviation (σ) of the Gaussian. This was followed by a binarization step with filling up of holes to avoid splitting ears during the watershed process that was performed with a tolerance of 40 (Fig. 4f). The number of ears was then computed from particles analysis after setting the minimum and maximum pixel area size (> 10 pixels^2^) to exclude anything that is not an object of interest in the image (https://imagej.nih.gov/ij/docs/guide/146-30.html#toc-Subsection-30.2). Ear length and width are referred here as the longest distance between two points along the major and minor axis on a single ear, respectively (Fig. 4f).

### Kernel count and kernel weight models

**Fig. 5:**
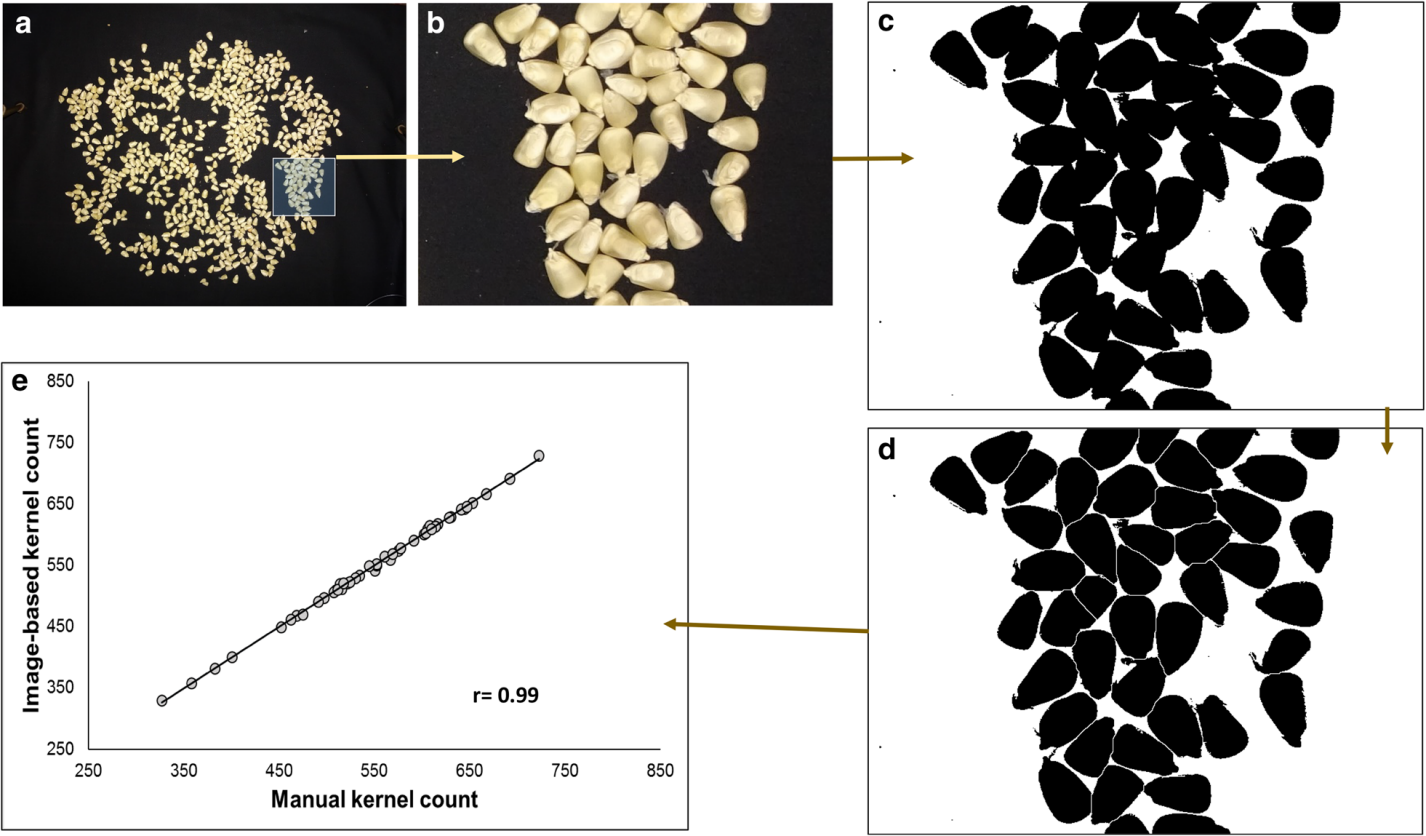
Example of image-based kernel count: **a** original image, **b** image section with many kernels in clusters, **c** transformation into 8-bit and binarization, **d** after adjustable watershed and **e** correlation between image-based kernel count and manual kernel count for 50 randomly selected ears
- To estimate the total number of kernel on a given ear from the number of kernels that are visible on a photo of the same ear, 340 ears were photographed individually using the same set up described before. The same ears were then threshed to remove their kernels which were put separately in paper bags and counted using the image-based kernel counting method describe above.

The development of a model to estimate the total number of kernels from photos of dehusked ears was done in two steps:

- To compare image-based kernel counting method with the manual kernel count, 50 randomly selected ears were threshed and their kernels put separately in paper bags. The kernels of each ear were first counted manually and then spread on a dark background and photographed using a camera (same set up as above). These images had numerous kernels in clusters (Fig. 5a, b). They were processed using imageJ plugins i.e. transformation into 8-bit, binarization, adjustable watershed with a tolerance of 3 and particle analysis (Fig. 5c, d). The correlation between the two methods was r = 0.99 (Fig. 5e). Therefore, the image-based kernel counting was considered as equivalent to the manual kernel counting method for kernels removed from ears.

A linear regression model for predicting total kernel number on an individual ears was developed from the number of kernels that are visible on the image (*kn*) (Eq. 2, r = 0.98***). The Pearson’s correlation coefficient r, was used to assess the relationship between estimated and measured kernel parameters.

2 $${\text{Total}}\,{\text{kernel}}\,{\text{number}} = 2.4051*kn - 6.7334$$

where *kn* is the number of kernels visible on the photo.

**Fig. 6:**
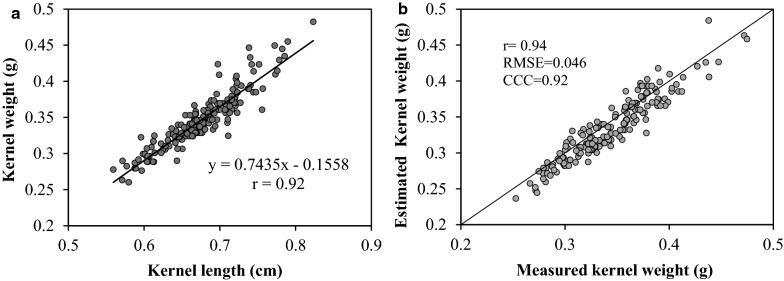
**a** Regression model for predicting kernels weight from kernel length and **b** validation of the kernels weight model (CCC = concordance correlation coefficient; RMSE = root-mean-square error and r = ρ = Pearson’s correlation coefficientfactor). (n = 200). The average kernel weight (total kernel weight divided by the total number of kernels) was measured manually using a digital balance while the average kernel length was extracted from the visible part of the segmented ear

The kernel weight model was developed using a linear regression model between average kernel length ($\overline{kl}$) and the average kernel weight (total kernel weight divided by the total number of kernels) measured manually using a digital balance (Mettler Toledo), at a precision of 0.01 g. Kernel weight was measured at a moisture content ranging from 11 to 13%. This was done using 200 ears with contrasting kernel size. The average kernel length was extracted from the visible part of the segmented ear. $\overline{kl}$ was plotted against the average measured kernel weight for each individual ear measured manually to develop a model that translates kernel length into kernel weight (Fig. 6a). The model was then tested and exhibited a quite accurate estimation of the kernel weight (Fig. 6b).

3 $${\text{Average}}\,{\text{kernel}}\,{\text{weight}}\,\left( g \right) = \left( {\overline{kl} *0.7435} \right) - 0.155$$

where $\overline{kl}$ is the average kernel length.

### Kernel weight estimation

Given that Eq. 2 provides the total kernel number and Eq. 3 the average kernel weight, the total kernel weight (Eq. 4) was computed as the product of these two equations:

4 $${\text{Total}}\,{\text{Kernel}}\,{\text{Weight}}\,\left( {\text{g}} \right) = (2.4051*kn - 6.7334)*(( \overline{kl} *0.7435) - 0.155)$$

The estimated total kernel weight was validated using plot level (2 rows plants, 34 plants) images acquired under field conditions from six different breeding trials.

### Data reliability test

Lin’s concordance correlation coefficient (CCC = ρ_c_) [22] was used to test the data reliability.

5 $$\uprho_{c} = \frac{{2\sigma_{12} }}{{\sigma_{1}^{2} + \sigma_{2}^{2} + (\mu_{1} - \mu_{1 } )^{2} }} = \rho C_{b}$$

where µ_1_ = E(Y_1_), µ_2_ = E(Y_2_), E = expected value, $\sigma_{1}^{2}$ = Var(Y_1_), $\sigma_{2}^{2}$ = Var(Y_2_), and σ_12_ = Cov(Y_1_, Y_2_) = σ_1_ σ_2_ ρ, C_b_ = 2 σ_1_σ_2_/[$\sigma_{1}^{2} + \sigma_{2}^{2} + (\mu_{1} - \mu_{1 } )^{2}$].

(ρ_c_) measures both precision (ρ) and accuracy (C_b_).

(ρ) = Pearson’s correlation coefficient, a measure of how close the data are about the line of best fit.

(C_b_) = Bias correction factor, a measure of how far a line of best fit (i.e. the line of perfect concordance) is from a 45 degree angle through the origin.

Lin’s coefficient is 1 when all the points lie exactly on the 45-degree line drawn through the origin and diminishes as the points depart from this line and as the line of best fit departs from the 45-degree line [23].

### Broad-sense heritability

The broad-sense heritability is the ratio of total genetic variance (V_G_) to total phenotypic variance (V_P_).

6 $${\text{H}}^{2} = {\text{ V}}_{\text{G}} /{\text{V}}_{\text{P}}$$

Broad-sense heritabilities were computed using Meta-R (multi environment trial analysis with R for windows) version 6.01 01 [24] and compared among traits for several field experiments.

Linear models were implemented using REML (restricted maximum likelihood) to calculate BLUEs (best linear unbiased estimations) and BLUPs (best linear unbiased predictions) and estimate the variance components.

 The broad-sense heritability of a given trait at an individual environment was calculated as:

7 $$H^{2} = \frac{{\sigma_{g}^{2} }}{{\sigma_{g}^{2} + \sigma_{e }^{2} /nreps}}$$

where *σ*_*g*_^2^ and *σ*_*e*_^2^ are the genotype and error variance components, respectively, and *nreps* is the number of replicates.

The genetic correlation between traits was calculated as:

8 $$\rho_{g} = \frac{{\overline{{\sigma_{{g\left( {jj^{\prime}} \right)}} }} }}{{ \overline{{ \sigma_{g\left( j \right)} \sigma_{{g\left( {j^{\prime}} \right) }} }} }}$$

where $\overline{{\sigma_{{g\left( {jj^{\prime}} \right)}} }}$ is the arithmetic mean of all pairwise genotypic covariances between traits *j* and *j′*, and $\overline{{ \sigma_{g\left( j \right)} \sigma_{{g\left( {j^{\prime}} \right)}} }}$ is the arithmetic average of all pairwise geometric means among the genotypic variance components of the traits.

The relationships between the image variables and reference measurements were tested for significant correlation using the Pearson correlation coefficient.

## Results

### Kernel count and ear attributes

**Fig. 7:**
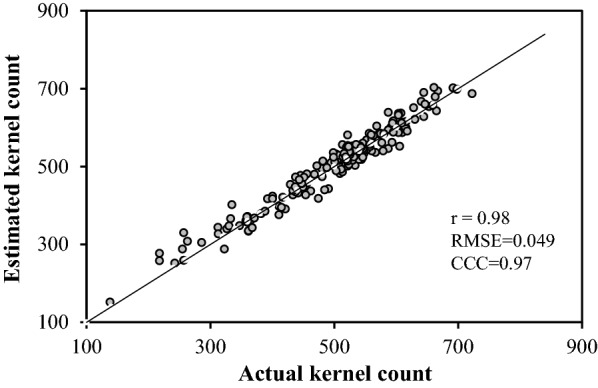
Validation of the kernel count model, (CCC = concordance correlation coefficient; RMSE = root-mean-square error and r = ρ = Pearson’s correlation coefficient)

**Fig. 8:**
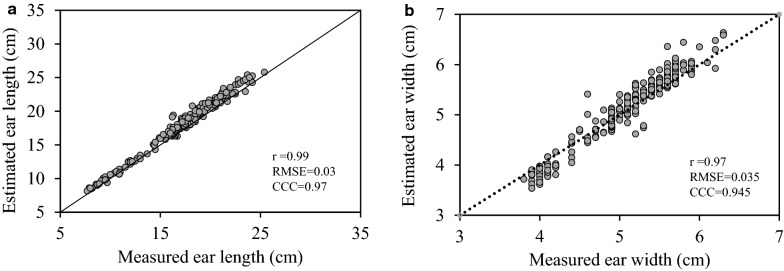
Relationship between measured and estimated ear **a** length and **b** width, (CCC = concordance correlation coefficient; RMSE = root-mean-square error and r = ρ = Pearson’s correlation coefficient)

The kernel count model was tested using 180 ears selected over a range of ear sizes from 150 plots as described in the methodology section. Data showed a linear correlation (r = 0.98, p < 0.001) between the estimated kernel count from intact ears using the model and the actual count of detached kernels (Fig. 7). The same ears used for kernel count validation were also used to compare manual measurements of ear length and width with those generated through the image processing method. Data presented a linear correlation (r > 0.98, p < 0.001) between the two methods for both traits (Fig. 8 a,b). A similar result was recorded for ear count that is much easier to do (data not shown).

### Kernel weight estimation

To validate the kernel weight estimation method, data were collected from six field trial (as described in the methodology). Measured kernel weight was compared with estimated kernel weight using the Lin’s concordance test. Results show that the values of the concordance correlation coefficient are all above 0.70 except for trials 2 and 4; with an average of 0.74 (Table 1). Average values of precision and accuracy were 0.88 and 0.83, respectively. This indicates that overall, the estimated kernel weight is in relatively good agreement with the measured kernel weight.

**Table 1:** Lin’s concordance correlation coefficient between measured and estimated kernel weight. Data are from six hybrid trials conducted under low soil nitrogen conditions at Harare, Zimbabwe, during the season 2016–2017

Trial	Number of plots	Concordance correlation coefficient	95% confidence interval	Pearson ρ (precision)	Bias correction factor C_b_ (accuracy)
1	150	0.7985	0.7434–0.8428	0.8573	0.9313
2	150	0.6281	0.5553–0.6913	0.8715	0.7207
3	150	0.7205	0.6447–0.7823	0.7754	0.9292
4	150	0.5969	0.5280–0.6580	0.906	0.6588
5	165	0.9243	0.9000–0.9429	0.9512	0.9718
6	165	0.7792	0.7394–0.8136	0.9744	0.7996
Mean	155	0.74125	–	0.8893	0.835233

### Heritability of kernel and ear attributes

Broad-sense heritability for measured grain yield averaged 0.44 across all trials, similar to that of estimated (Table 2) total kernel weight and total ear area, but significantly lower if compared to the heritability of kernel size (average length and width, average area and perimeter) and to a lesser extent the total kernel number (Table 2). The number of ears per plot and the average ear length had higher heritability than measured grain yield, which is not the case of average ear width.

**Table 2:** Broad-sense heritabilities (*H*^*2*^) and means for grain yield and kernel/ear attributes estimated through imaging for six maize trials with three replicates evaluated under low soil nitrogen at Harare, Zimbabwe

Trial	Number of		Measured Grain yield(Mg ha^−1^)	Broad-sense heritability *(H*^*2*^*)*										
				Kernel attributes								Ear attributes		
	Entries (hybrids)	Year		Visible Kernel Number	Mean width (cm)	Mean length (cm)	Total area (cm^2^)	Mean area (cm)	Mean perimeter (cm)	Total Number per plot	Total Weight (g plot^−1^)	Number per plot	Mean length (cm)	Mean width (cm)
EHYB1746	50	2017	0.591	0.374	0.725	0.815	0.439	0.71	0.842	0.374	0.301	0.781	0.665	0.507
EHYB1747	50	2017	0.596	0.619	0.657	0.761	0.513	0.722	0.765	0.619	0.492	0.358	0.728	0.634
EHYB1748	50	2017	0.595	0.624	0.709	0.624	0.687	0.69	0.569	0.597	0.700	0.746	0.539	0.278
IHYB1747	50	2017	0.599	0.721	0.737	0.693	0.423	0.607	0.735	0.721	0.442	0.515	0.652	0.504
LHYB1619	55	2016	0.146	0.541	0.904	0.930	0.238	0.917	0.934	0.541	0.320	0.647	0.560	0.730
LHYB1617	55	2016	0.137	0.314	0.798	0.830	0.384	0.782	0.903	0.314	0.287	0.450	0.279	0.239
Mean			0.444	0.532	0.755	0.775	0.447	0.738	0.769	0.527	0.423	0.582	0.570	0.482
				Mean										
EHYB1746	50	2017	4.02	4510.01	0.36	0.66	760.64	0.17	1.85	9243.27	3001.43	24.51	14.95	4.81
EHYB1747	50	2017	5.48	5337.80	0.35	0.65	858.42	0.16	1.81	10941.06	3452.16	28.22	14.71	4.70
EHYB1748	50	2017	2.60	4781.87	0.36	0.67	801.47	0.14	1.91	9800.89	3237.06	27.88	14.42	4.66
IHYB1747	50	2017	5.20	5398.55	0.34	0.63	824.45	0.15	1.77	11065.71	3318.40	28.05	14.34	4.64
LHYB1619	55	2016	2.24	3789.83	0.42	0.76	852.04	0.23	2.31	7766.21	2415.55	21.77	16.43	5.23
LHYB1617	55	2016	1.36	3151.16	0.35	0.60	435.25	0.14	2.02	4420.99	1126.73	23.18	11.17	3.93

*EHYB* early hybrid trial, *IHYB* intermediate hybrid trial, *LHYB* late hybrid trial

## Discussion

Maize grain yield can be described as a function of the number of harvestable kernels and their individual weight. From these two yield determinants, kernel number usually explains most variation [25] and is strongly related to ear size. Several studies have reported that kernel weight is a highly heritable trait [26, 27], varying markedly among genotypes [28] and largely influenced by genotype × environment interactions. Maize kernel weight is associated with the duration of the grain-filling period, the rate of kernel biomass accumulation, the rate of kernel desiccation and the moisture concentration at physiological maturity [29]. All these traits had large phenotypic variation and significant response to the interaction between genotype and environment [30]. Although very important, kernel traits are not easy to measure rapidly and accurately, partly due to the need for ear threshing before they can be measured. Kernel count can be done manually by counting the number of rows and multiplying that by the number of kernels in one length of the ear. Regarding ear number and size, the manual methods of data collection include measuring directly the dimensions of an individual ear or kernel with calipers [17]. These manual measurements of yield components have been useful and were, for example, used for a divergent selection study of the relationship between ear length and yield [31]. The problem with these methods is the lack of consistency that is inherent to the way the data is collected (dependent on the training and appreciations of the staff devoted to that task), the time and associated cost, which makes them mostly suitable for very small trials. From a preliminary assessment (data not shown), the proposed EDI method can be twice (example: ear count) to five-fold (example: ear dimensions) or more, faster than the manual methods depending on the targeted measurement. The manual methods are labor intensive, which makes them costly as compared to the EDI method. The difference in terms of cost would depend on the location/country because of variations in the cost of labor. Yield component studies as well as selection for crop improvement could take advantage of automated measurements that are more consistent, fast and low-cost. For example, Takanari et al. [32] and Moore et al. [33] mapped quantitative trait loci (QTL) in rice and Arabidopsis, respectively using image-derived size and shape phenotypes.

Miller et al. [1] have proposed an imaging method of kernel counting based on individual kernel area. The method estimates also kernel size (width and depth) but only on detached kernels. While this method is quite precise, it requires that the kernels are removed from the ears; which may not be convenient especially when dealing with a large number of ears. Similarly, Liang et al. [14], have also developed a method that scores maize kernel traits based on line-scan imaging that cannot be suitable for assessment in the field in terms of time and cost. The advantage of the proposed EDI method it that it generates ear and kernel attributes data from images of intact ears. The approach is to some extent similar to that of Grift et al. [34] who have developed a machine vision-based method to count maize kernels on the ear within a quasi-cylindrical mid-section and ear maps. While their method is, to a large extent, interesting; the imaging is done in a soft box fitted with a light reflector and high-quality diffused lighting scene. The limitation of this type of imaging set up is the throughput. Regarding ear size, the EDI method showed a good agreement between manually measured ear dimensions and the results of automated image processing (Fig. 8). Similar results were reported by Miller et al. [1]. The main difference between the two methods is that the one proposed by Miller et al. [1] uses flatbed document scanners to acquire ear images whereas the EDI method makes use of RGB camera. In addition, while the flatbed scanner gives the advantage of controlling lighting conditions; the logistics associated with using it in the field (i.e. need of a computer) and the limited number of ear (3–5) that can be scanned at a time does not make it suitable for assessing thousands of ears that are usually evaluated in a breeding trial.

The EDI method also estimates kernel weight through kernel size, thereby providing an opportunity for a cheap yield performance assessment, especially in case where ear shelling and kernel weighing may be too costly or the required equipment not available. It is important to mention that this method does not systematically take into account kernel moisture (the kernel weight model was developed for a range of kernel moisture between 11 and 13%), which often quite significantly affect the actual weight if not corrected for. In addition, the EDI method does not include kernel depth for weight estimation which in some cases may lead to a slight underestimation of the actual kernel weight.

### Factors affecting extraction of kernel attributes *(color, texture and surface reflectance)*

**Fig. 9:**
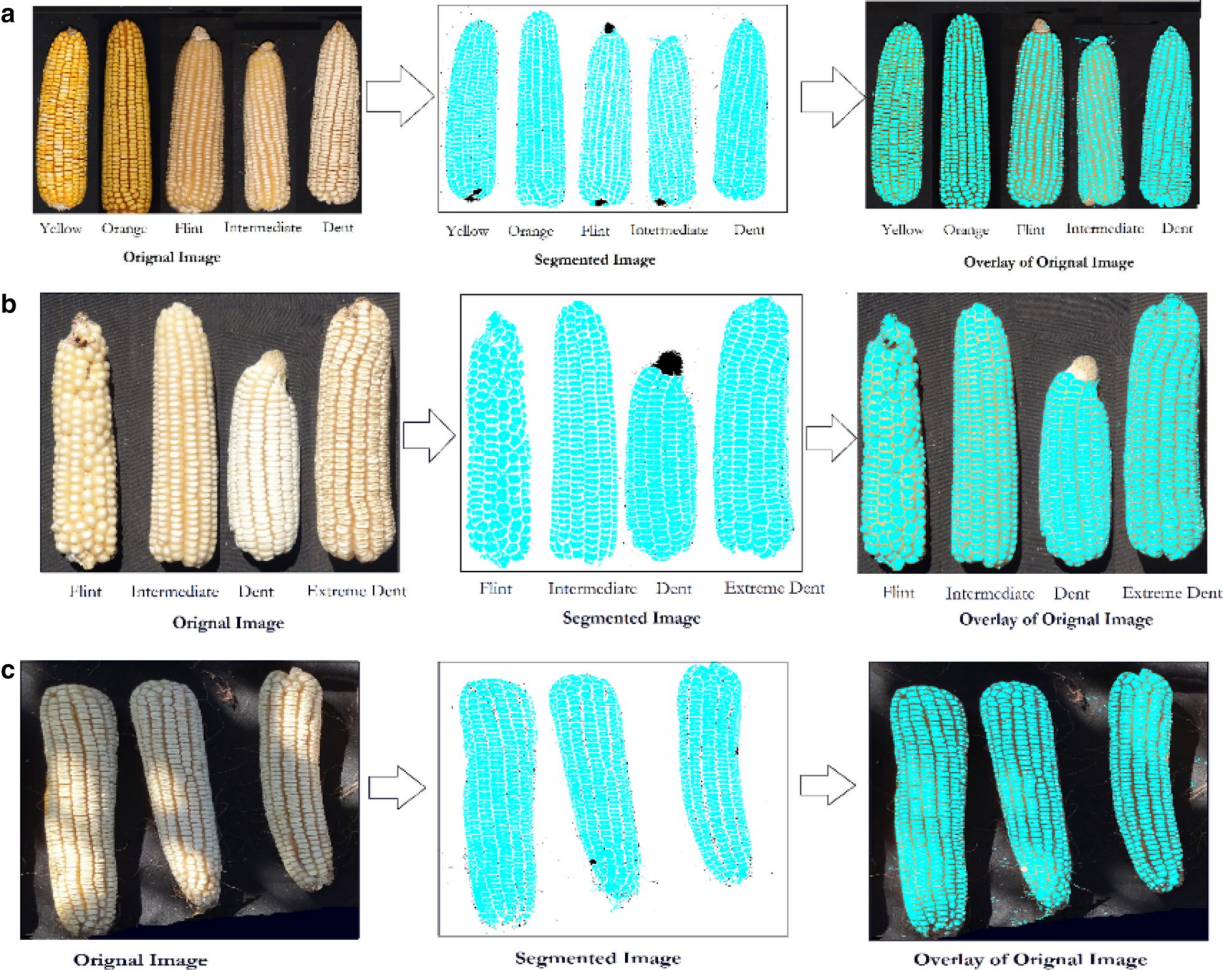
Maize ears with different (**a**, **b**) color, texture and size taken under open light and field conditions. **c** shading effect

 Maize ears are diverse in color and texture. The proposed method was tested on different ear colors and textures. As shown in Fig. 9a, b, ears were successfully segmented across tested colors and sizes. However, ears with flint kernels showed underestimated kernel size as compared to those with dent kernels (data not shown). This is largely because most flint kernels are multi-colored in addition of concave surfaces surrounded by wide and hazy boundaries which negatively affect the segmentation process. On the other hand, with dent ears, which have uniformly white and flat surfaces segments, kernels are much easier to segment.

Besides, kernel color and texture, lighting conditions can constitute a challenge for image processing, largely due to surface reflections. This can affect both the kernel count and size estimation because these reflections affect the quality of color segmentation. The proposed method showed a relatively good segmentation for ears that have kernel surface reflections due to non-uniform lighting conditions (Fig. 9c).

## Conclusion

This work has shown that the EDI method can be used as an alternative to the traditional methods of ear phenotyping. It is more consistent than manual measurements, which typically employ calipers and manual counting especially for large number of ears that are often evaluated in breeding trials. The accuracy of this method rely largely on the resolution of the camera that is used; however this does not represent a major challenge because of the recent significant improvement in the resolution of all camera types, including those of smartphone or tablet.

From a breeding perspective, kernel number, their total area and weight and number of ears generated through the current method could be a valuable adjunct in increasing the efficiency of selection for grain yield due to their genetic correlation with grain yield and relatively high broad-sense heritability combined with low selection cost. The method will be particularly helpful for breeding programs that have limited operational resources. The ability to measure ear and kernel attributes together may help to develop varieties with desirable farmers preferred traits like ear or kernel size.
