# Correction to: High-throughput method for ear phenotyping and kernel weight estimation in maize using ear digital imaging

**DOI:** 10.1186/s13007-019-0431-y

**Published:** 2019-05-22

**Authors:** R. Makanza, M. Zaman-Allah, J. E. Cairns, J. Eyre, J. Burgueño, Ángela Pacheco, C. Diepenbrock, C. Magorokosho, A. Tarekegne, M. Olsen, B. M. Prasanna

**Affiliations:** 1 International Maize and Wheat Improvement Center (CIMMYT), PO Box MP163, Harare, Zimbabwe; 2 University of Queensland, Brisbane, Australia; 3 International Maize and Wheat Improvement Center (CIMMYT), PO Box 1041, Nairobi, Kenya; 4 International Maize and Wheat Improvement Center (CIMMYT), El Batan, Mexico; 5 Cornell University, Ithaca, NY 14853 USA

## Correction to: Plant Methods (2018) 14:49 10.1186/s13007-018-0317-4

After the publication of our article [1], it was brought to our attention that in six places in the article we omitted to use quotation marks to show where the text has been directly used from the cited references. The sentences which should be corrected are shown below. All reference numbers shown in this erratum text are those used in the original article.

Original text (Background section):

Cairns et al. [3] reported that under drought conditions, yield loss in both hybrids and inbreds was largely associated with a highly significant decrease in the number of kernels per unit of ear area.

Should read:

Cairns et al. [3] reported that under drought conditions, “yield loss in both hybrids and inbreds was largely associated with a highly significant decrease in the number of kernels per unit area”.

Original text (Background section):

From a breeding perspective, studies have found that yield components tend to display greater heritability than overall yield [9, 10].

Should read:

From a breeding perspective, “studies have found that yield components tend to display greater heritability than overall yield” [9, 10].

Original text (Background section):

According to Miller et al. [1], if maize ears, and kernels attributes could be automatically measured with greater objectivity and precision, more could be learned about the genetic bases of yield components and how to improve them using current and future maize genetic resources.

Should read:

According to Miller et al. [1], “if maize ears, cobs, and kernels could be automatically measured with greater objectivity and precision, more could be learned about the genetic bases of yield components and how to improve them using current and future maize genetic resources.”

Original text (Background section):

In addition, Miller et al. [1] have proposed three custom algorithms designed to compute kernel features automatically from digital images acquired by a low cost platform. One algorithm determines the average space each kernel occupies along the cob axis using a sliding-window Fourier transform analysis of image intensity features. The second one counts individual kernels removed from ears, including those in clusters. The third one measures each kernel’s major and minor axis.

Should read:

In addition, Miller et al. [1] have proposed three custom algorithms designed to compute kernel features “automatically from digital images acquired by a low‐cost platform. One algorithm determines the average space each kernel occupies along the cob axis using a sliding‐window Fourier transform analysis of image intensity features. A second counts individual kernels removed from ears, including those in clusters. A third measures each kernel’s major and minor axis” [1].

Original text (Materials and methods section):

Lin’s coefficient is 1 when all the points lie exactly on the 45-degree line drawn through the origin and diminishes as the points depart from this line and as the line of best fit departs from the 45-degree line [23].

Should read:

“Lin’s coefficient is 1 when all the points lie exactly on the 45-degree line drawn through the origin and diminishes as the points depart from this line and as the line of best fit departs from the 45-degree line” [23].

Original text (Discussion section):

All these traits had large phenotypic variation and significant response to the interaction between genotype and environment [30].

Should read:

“All these traits had large phenotypic variation and significant response to the interaction between genotype and environment” [30].

The authors apologise for this oversight.
